# Observation of terahertz-radiation-induced ionization in a single nano island

**DOI:** 10.1038/srep10280

**Published:** 2015-05-22

**Authors:** Minah Seo, Ji-Hun Kang, Hyo-Suk Kim, Joon Hyong Cho, Jaebin Choi, Young Min Jhon, Seok Lee, Jae Hun Kim, Taikjin Lee, Q-Han Park, Chulki Kim

**Affiliations:** 1 Sensor System Research Center, Korea Institute of Science and Technology, Seoul 136-791, Republic of Korea; 2 Department of Physics, Korea University, Seoul 136-701, Republic of Korea

## Abstract

Terahertz (THz) electromagnetic wave has been widely used as a spectroscopic probe to detect the collective vibrational mode in vast molecular systems and investigate dielectric properties of various materials. Recent technological advances in generating intense THz radiation and the emergence of THz plasmonics operating with nanoscale structures have opened up new pathways toward THz applications. Here, we present a new opportunity in engineering the state of matter at the atomic scale using THz wave and a metallic nanostructure. We show that a medium strength THz radiation of 22 kV/cm can induce ionization of ambient carbon atoms through interaction with a metallic nanostructure. The prepared structure, made of a nano slot antenna and a nano island located at the center, acts as a nanogap capacitor and enhances the local electric field by two orders of magnitudes thereby causing the ionization of ambient carbon atoms. Ionization and accumulation of carbon atoms are also observed through the change of the resonant condition of the nano slot antenna and the shift of the characteristic mode in the spectrum of the transmitted THz waves.

Recently, nonlinear interaction of strong THz radiation with matter has become a reality^1^ through the development of various techniques for generating intense terahertz (THz) field^2^,^3^,^4^,^5^,^6^. Nonlinear THz studies are important not only for understanding fundamental light-matter interaction, but also for providing possible applications in nonlinear THz optics^7^. The nonlinear conditions include the optical Kerr effect^8^, in which the change in a material’s refractive index can be induced by a single cycle of THz pulses, and the dynamical Franz–Keldysh effect^9^, wherein the band structure of a semiconductor is modified by an intense electric field or a strong impact ionization effect^10^. Nonlinearity in THz frequencies is realized via the acceleration of charged carriers under the intense THz electric field and the resulting large kinetic energies of charge carriers proportional to the half cycle of the field. Although it is generally expected that THz light is a non-ionizing source, since the photon energy (~4 meV at 1 THz) at THz frequencies is not enough to ionize atoms or molecules, strong THz sources can cause a greatly enhanced nonlinear absorption and even field ionization^11^,^12^. For example, the ionization of Na atoms in high-lying Rydberg states by intense half-cycle THz electromagnetic pulses has been also demonstrated^13^.

Here, we report the observation of THz radiation induced ionization of ambient carbon atoms in a uniquely designed metallic nano structure. The structure, composed of a nano slot antenna and a nano island located at the center, confines THz electromagnetic waves at the center, resulting in huge local field enhancement by orders of magnitude^14^. The metallic nano island inside a nano slot effectively narrows down the gap size and generates local fields strong enough to trigger the ionization of carbon atoms^15^. The field emitted electrons from the surrounding metal surface can ionize the ambient carbon atoms via the inelastic electron scattering. In addition, the direct field ionization is considered to play a role in the process^13^. The accumulated carbon atoms form a layer of a carbon composite material around the nano island. The resonance frequencies of the transmitted THz field are determined by geometrical conditions of the system^16^. We observe that the fundamental resonance mode in the THz spectrum is shifted to the first excited mode due to such a conformational change of the system. Our uniquely designed nanostructure has a great advantage for engineering the THz field enhancement and the ability to trigger the ionization of ambient carbon atoms even under a medium strength THz radiation of 22 kV/cm, that has not be shown in earlier works.

A metallic nano island 160 nm in diameter was placed in a 200-nm-wide and 70-μm-long slot antenna that was fabricated onto a 500-μm thick Si wafer, as shown in Fig. 1(a). The nano island and slot antenna were defined by focused ion beam (FIB) after deposition of a 100-nm-thick gold layer and a 10-nm-thin Ti layer for adhesion on top of the Si substrate. A CF_4_ plasma etch step was then applied to mill down the Si substrate around the nano island inside the slot antenna. The gold layer withstood the etching process, resulting in a metallic island on top of the Si nano pillar that was completely isolated and electrically disconnected from the slot antenna (Fig.1(b)). Transmitted THz field through the sample was measured using the THz time domain spectroscopy system with a second-order nonlinear optical medium of (110) ZnTe for both generation and detection of the THz field (Fig. 1(c)). Details of the experimental conditions are further explained in the methods section (see below).

The fundamental finding of our work is shown in Fig. 2(a) where the measured THz transmission through the slot antenna with the gap distance of 200 nm is plotted for the cases of with and without the metallic nano island under various strength THz field of 18.3 ~ 22 kV/cm. The amplitude of a transmitted wave, *T*, is normalized by the one through a void aperture: *T* = *E*_*sample*_*/E*_*ref*_. The fundamental resonance frequency through the slot antenna only is 0.67 THz (black dotted) related to the length of the slot. In the nano island embedded slot measurements, as the incident THz field strength increases, the resonance peak in the spectrum is shifted towards higher frequencies. It is noted that progressive shifted resonance frequency at 1.0 THz (green, 18.3 kV/cm) and 1.14 THz (orange, 19.0 kV/cm), respectively, reflect the mid-process of carbon layer growth. These intermediate resonance modes were reported as continuous transition states between full- and half-wavelength states with various thickness of nano metal barriers^17^. Microscopic interpretation of the transition can be made by taking into account the charge transfer between two rims of the slot. In this point of view, deposited carbon layer acts as a bridge allowing the charge transfer: the more deposition, the easier charge transfer will occur. In order to mimic the transition of the resonance depending on the degree of the charge transfer, we performed a finite-difference time-domain (FDTD) method based simulation^14^. We assumed that there is a metal-like medium of negative permittivity filled inside the narrow gap between the nano island and the slot antenna, and controlled the degree of the charge transfer by changing the permittivity of the bridge with several exemplary values between –3,000 and –40,000. The obtained transmission spectrum in Fig. 2(b) shows that the resonance progressively shifts from full-size state to nearly half-size state as the magnitude of the permittivity increases. The trend of transition is quite similar to that of measured spectra in Fig. 2(a), reflecting that a stronger THz power yields thicker deposition of the carbon layer. As we will discuss later, our nano-island enables the carbon deposition by increasing local THz field dramatically. The enhanced THz field amplitude around a nano island in a slot antenna is calculated using the FDTD method. The obtained spectrum in the inset of Fig. 2(b) shows a field enhancement factor of about 800 at 0.67 THz, which is the fundamental resonance mode of the slot antenna. This outstanding field enhancement implies that our uniquely designed system has a merit of an additional enhancement from the proximity of the metallic nano island with the slot antenna. It should be emphasized that the field enhancement reported above is very unusual considering that the calculated field enhancement factor for a 20-nm-wide slot antenna is about 300 under the same calculation. For further applications, one can take embedding an island at certain point inside the antenna into account, in terms of controlling the enhancement point freely, meanwhile the enhancement point is fixed at a center in simple slot antenna structure. After the exposure to the external THz wave, we observed in the SEM image that the nano slot antenna is covered by a layer of carbon composite material especially at the center, bridging the gap between the island and the slot antenna (Fig. 2(c)). In contrast, the edge of the nano slot antenna remained undisturbed by the carbon contamination (Fig. 2(d)), manifesting that the enhanced field was highly focused at the center of the slot antenna. It can be explained that the resonance spectrum of the transmitted THz waves was shifted to the first excited mode 1.37 THz (frequency doubled) by the newly formed carbon layer making the slot antenna half its original length^18^.

Our device structure was designed such that the enhanced THz field in a specific spot easily exceeds the usual field amplitude provided by conventional THz wave sources (on the order of several MV/cm). The highly enhanced and confined electric field causes the accumulation of charges and introduces a lambda-zone around the metal slot^19^, as schematically drawn in Fig. 3(a). This results in 800 times increased amplitude of the THz electric field (640,000 times larger in intensity) at the center of the slot antenna. Based on the relative transmission, the maximum amplitude of the enhanced field is estimated as 17 MV/cm. This high field THz source allows *non-resonant* THz control over matter. That is, the high THz field can accelerate electrons in the carbon atoms and on the surface of the metal over half a cycle of the field, exciting electrons due to the relatively long sustainability of the THz field. There have been previous researches in which bounded electrons can be excited in different materials under strong THz waves^1^,^7^,^20^,^21^. Considering the work done by the THz field on an electron over the half cycle of the radiation, the ponderomotive energy^22^ can be estimated as,


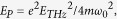


where *e* is the electron charge, *E*_*THz*_ is the peak amplitude of the THz field, *m* is the electron mass and *ω*_*0*_ is the frequency of the THz field. Unlike resonant light coupling with electronic and ionic states that cause the phase-coherent accumulation of the incident energy, control via a nonresonant field can take place once the stored energy via the acceleration of electrons meets the certain threshold energy for a corresponding process. Electrons under the control of a 0.7 THz wave (the fundamental resonance frequency of our system) with a peak field of 17 MV/cm reach a maximum kinetic energy of 3,500 eV, which greatly exceeds the typical range of the work function for gold (5.1 ~ 5.47 eV) and the ionization energy of carbon atoms (11 eV). The dry etching process with CF_4_ plasma, which extends the nano island into a pillar structure, plays a crucial role in lowering the work function of the gold surface and increasing the emission rate as reported elsewhere^23^. Therefore, electrons out of the gold surface have enough energy to ionize the ambient carbon atoms in the vacuum chamber and even carbon atoms can be directly ionized by the THz electric field^13^. Those ionized carbon elements are deposited on the surface along the field distribution. This newly formed carbon layer prevents accumulation of the charges around the metallic nano slot (schematically drawn in Fig. 3(b)), since now the coated carbon layer electrically connects the island-to-slot^24^,^25^. Carbon contamination under high electric fields has been introduced previously in a corona discharge^26^, in field electron emission by Si nano-pillars^27^, and for fabrication of nano-tips^28^. However, to the best of our knowledge, this is the first observation of the ionization of atoms induced by incident THz wave by using plasmonics nano structures.

To validate the experimental result, we computed the THz field distribution around a nano island in a slot antenna using a FDTD method. We note that the distribution of the electric field amplitude inside a nano slot of length, *l*, perforated in a highly conducting metal film can be described by the excitation of the system in the fundamental mode, *E*_*x*_ = *A* sin(*πy/l*), where *A* is a proportionality constant. With the excitation of the fundamental mode, the slot antenna supports the maximum amplitude of the electric field at its center (*y* = *l*/2) and suppresses the field at both edges (*y* = 0, *l*). The electric field distribution in resonance at 0.7 THz is well simulated from the calculation based on the real parameters. A zoomed-in view of the field distribution at the center and the edge are shown in Fig. 3(c,d). Interestingly, the top view of the nano slot antenna after the exposure shown in Fig. 3(e,f) has the same feature as the calculated electric field distribution, reflecting that the deposition of the carbon layer is critically affected by the enhanced THz field. From composition analysis on the surface by Auger electron microscopy as shown in Fig. 3(g,h) (see methods), it is confirmed that the newly formed layer around the nano island is mainly composed of carbon.

A crucial test for the enhanced THz field and the induced ionization in a designed device is carried out by controlling the geometric parameters in the nanostructures. Two different samples were prepared: one has an island-to-slot distance of 35 nm (a 130 nm diameter island embedded in a 200-nm-wide slot antenna) (Fig. 4(a)) and the other one has an island-to-slot gap of 80 nm (a 270 nm diameter island embedded in a 430-nm-wide slot antenna) (Fig. 4(b)). The transmissions were measured at various THz strengths in the range of 18.3 ~ 22.0 kV/cm. As the THz strength increases, the resonance peak shifts to higher frequencies as in 20 nm gap sample (see Fig. 2(c)). The resonance shift, however, is saturated at 1.16 THz in the applied maximum THz strength. It is seen in the SEM image that the carbon contamination is less than the one with 20 nm gap sample. In stark contrast, the measured field amplitude in the resonance spectrum slightly increases, but, the fundamental resonance frequency at 0.75 THz does not change significantly for 80 nm gap sample (Fig. 4(b)). The field enhancement factor is estimated to be much less than the one for the 20 nm island-to-slot gap structure. No prominent physical change after the THz exposure was observed in the SEM images as well (see Fig. 4(c,d)). The electric field dependence of resonance shift at different gap distances is summarized in Fig. 4(e). This tells us that the enhancement factor for ionization threshold is determined by both island-to-slot gap width and incident THz field strength.

In conclusion, we have fabricated a nano slot antenna in which placing a metallic nano island in a slot antenna enhanced the THz electric field amplitude significantly in a highly confined spot. A THz time domain spectroscopy system operating in a vacuum chamber was applied for the source and detection of the THz wave. It was observed that after the THz radiation on the sample, a layer of carbon composite material was deposited along the enhanced electric field and such a structural change of the nano slot antenna was reflected in the transmission spectrum. The ionization of ambient carbon atoms was realized with a metallic nanostructure under the radiation of a medium strength THz radiation. Nano slot antennas with different island-to-slot distances were also examined and the THz power dependent resonance frequencies were investigated. Our observation of THz field induced carbon ionization in the specially designed nanostructure shows a possibility for a new means of controlling matter and light in the atomic scale. These efforts utilizing nano-structures and nano-materials in the field of nonlinear THz radiation will finally lead to further advances in THz technology and diverse applications in the near future.

## Methods

### High power terahertz spectroscopy

A regenerative amplifier laser beam, with 1 kHz repetition rate providing 35 fs and 3 mJ pulses centered at 800 nm, is irradiated on a ZnTe crystal to produce intense THz radiation with the peak-to-peak power between 18.3 ~ 22 kV/cm using a variable filter. The generated THz wave with a horizontal polarization is focused by two Tsurupica lenses with a focal spot size of 2 mm and is incident on the slot antenna. The THz radiation is focused again onto the ZnTe crystal for detection. In order to capture the transmitted THz field through the sample, a balanced detection system for electro-optic sampling is employed. The entire THz spectroscopy system is placed in a vacuum chamber (10^−2^ ~ 10^−3^ Torr) and all measurements are performed at room temperature.

### Finite-difference time-domain calculation

For the numerical calculations, we used the finite-difference time-domain (FDTD) method with a grid size of 5 nm. The dispersive nature of the metal was implemented using the Drude model and the auxiliary differential equation method.

### Auger electron spectroscopy and microscopy

A Nanoprobe system (Model: PHI-700 scanning AES nanoprobe, ULVAC-PHI, INC) is used for non-destructive analysis to identify and quantify the chemical elements in the top few atomic layers of the sample surface. A focused electron beam examines the surface of the target sample and the emitted Auger electrons are collected in an electron energy analyzer. The electron beam energy is 5 kV and the target current is 10 nA (for mapping) with a 500 eV sputtering rate.

## Author Contributions

M.S. developed the concept, carried out the THz measurement in collaboration with H.-S.K., J.C., and Y.J. and wrote most of the paper. C.K. also wrote a part of the paper, and designed and made the samples with J.-H.C. J.-H.K. calculated spectrum using FDTD method and carried out analysis of data with Q.-H.P. S.L., J.H.K. and T.L. contributed to analyze experimental data and prepared figures. All authors discussed the results, and commented on and edited the manuscript.

## Additional Information

**How to cite this article**: Seo, M. *et al.* Observation of terahertz-radiation-induced ionization in a single nano island. *Sci. Rep.* **5**, 10280; doi: 10.1038/srep10280 (2015).

## Figures and Tables

**Figure 1:**
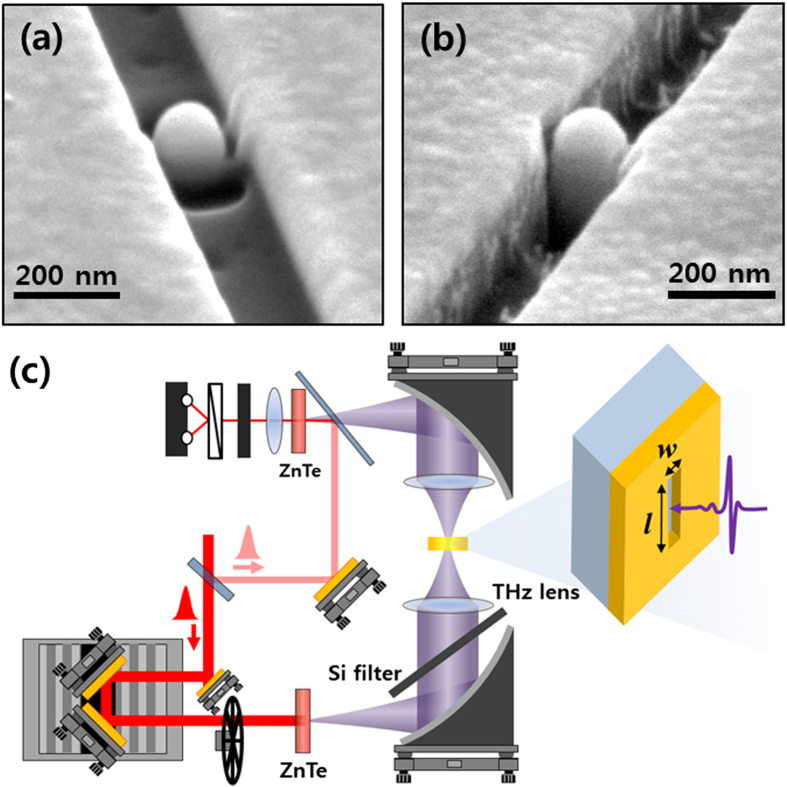
A nano island embedded in a slot antenna sample and high power time domain THz system. Scanning electron microscope (SEM) images of (**a**) a metallic nano island in a slot antenna defined by a focused ion beam (FIB) and (**b**) a nanopillar structure with a gold island on top formed by a reactive ion etching (RIE) process. (**c**) Schematic of a high power THz source and detection system. Amplified femtosecond laser pulses with a 1 kHz repetition rate were applied for the THz time domain spectroscopy.

**Figure 2:**
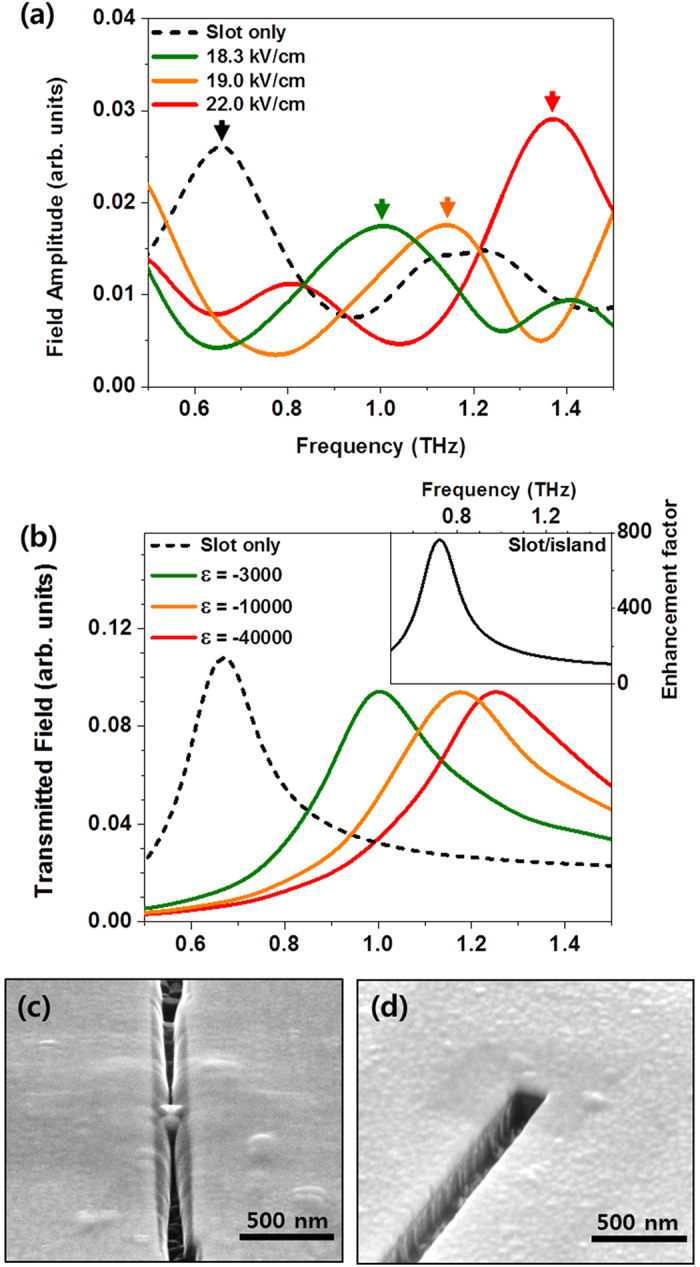
Spectrums of transmitted THz field amplitudes by strong THz field irradiation at a narrow island-to-slot antenna structure and SEM images. (**a**) THz power dependent transmitted spectrums for a 200-nm-wide slot antenna with (red solid line) and without (black dashed line) a 160-nm-diameter metallic island at the center. THz field strength is varied between 18.3 ~ 22.0 kV/cm. (**b**) A transmitted field amplitudes were calculated at the slot only (black), the island-to-slot gap filled with permittivity of -3,000 (green), -10,000 (orange), and -40,000 (red) using the finite-difference time-domain (FDTD) method. The inset represents calculated enhancement factor for island-to-slot gap distance of 20 nm using the same method. SEM images represent that carbon contaminant is formed around the nano island after the THz irradiation: the newly formed layer bridges the nano gap across the slot antenna (**c**), and both edges of the nano slot antenna remain clean (**d**), showing that the enhanced field is particularly focused around the nano island position.

**Figure 3:**
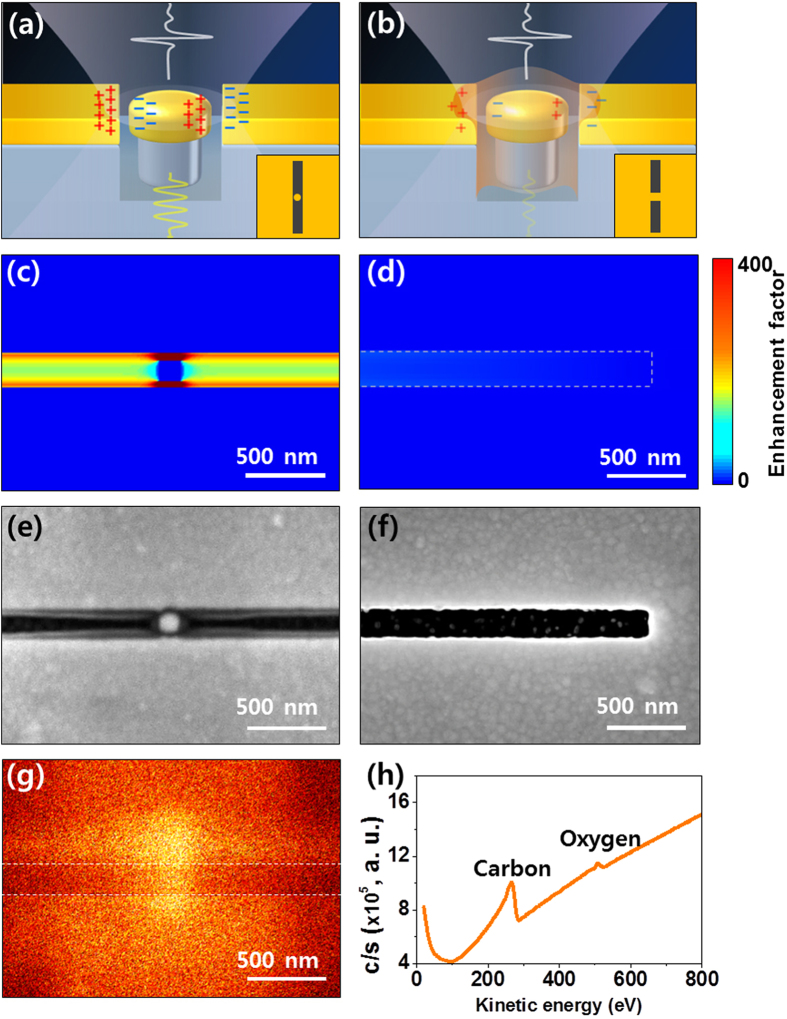
Schematics and images of charge distribution around the nano island. (**a**) Charge accumulation induced by the incident THz wave according to the local capacitor model^24^. (**b**) A newly formed carbon layer electrically connecting the gap between the slot antenna and the nano-island. Inset image describes a top view of each sample. The distributions of the THz electric field around the nano island (**c**) and at the edge of the slot antenna (**d**) are similar to the top view of the carbon deposited island (**e**) and the edge of the slot antenna without carbon (**f**). An Auger microscopy image represents the distribution of carbon elements around the island as shown in (**g**). (**h**) The carbon elements are confirmed with Auger spectroscopy performed around the nano island.

**Figure 4:**
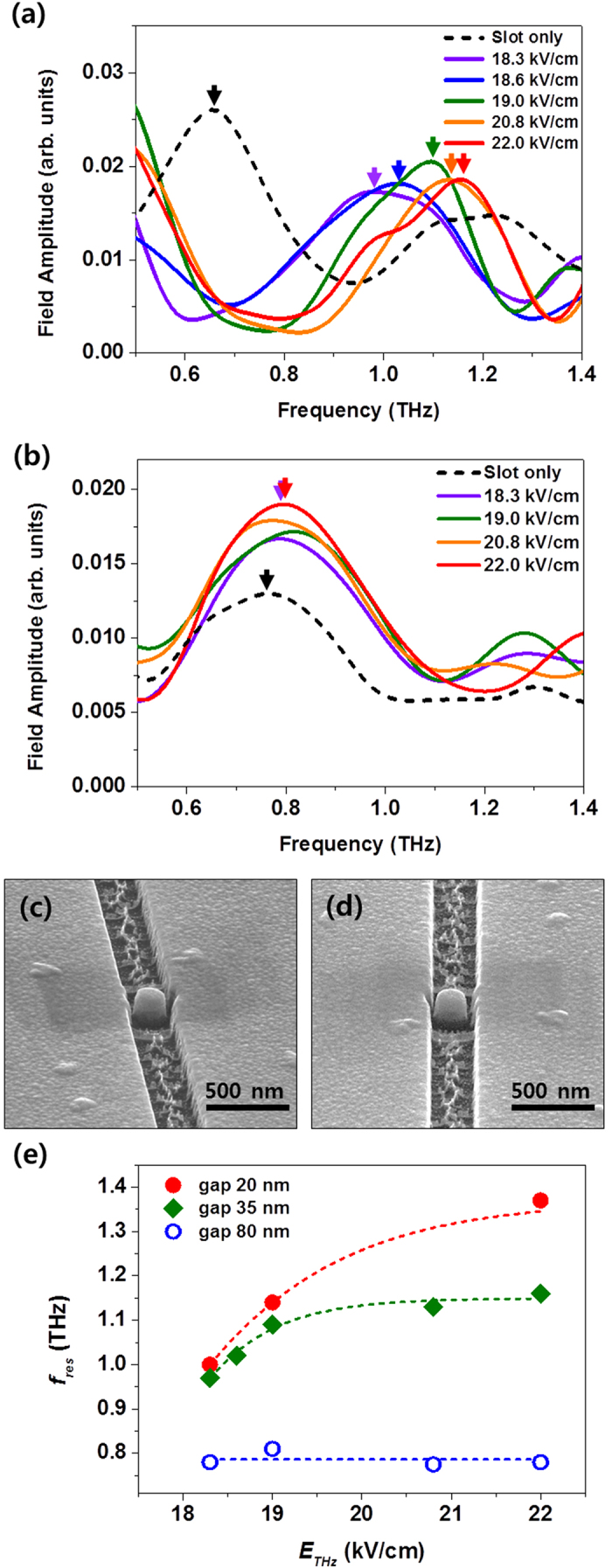
THz spectrums and sample geometry by strong THz field irradiation at several island-to-slot antenna structures. Transmission spectrums via two slot antennas: one with an island-to-slot gap distance of 35 nm (**a**) and the other one with an island-to-slot gap distance of 80 nm (**b**) measured under THz field strengths of 18.3 ~ 22.0 kV/cm. SEM images of the slot antenna with the nano island (island-to-slot gap distance of 80 nm) before (**c**) and after (**d**) the THz radiation represent that the structure with a 80 nm gap distance is unhampered by THz field radiation. (**e**) THz resonance frequencies (*f*_*res*_) for three different island-to-slot gap widths are shown in terms of THz field strengths (*E*_*THz*_).
